# Review: Corneal epithelial stem cells, their niche and wound healing

**Published:** 2013-07-24

**Authors:** Federico Castro-Muñozledo

**Affiliations:** From the Department of Cell Biology, Centro de Investigación y de Estudios Avanzados del IPN, México City, México

## Abstract

Stem cells emerged as a concept during the second half of 19^th^ century, first as a theoretical entity, but then became one of the most promising research fields in cell biology. This work describes the most important characteristics of adult stem cells, including the experimental criteria used to identify them, and discusses current knowledge that led to the proposal that stem cells existed in different parts of the eye, such as the retina, lens, conjunctiva, corneal stroma, Descemet’s membrane, and the subject of this review: the corneal epithelium. Evidence includes results that support the presence of corneal epithelial stem cells at the limbus, as well as the major obstacles to isolating them as pure cell populations. Part of this review describes the variation in the basement membrane composition between the limbus and the central cornea, to show the importance of the corneal stem cell niche, its structure, and the participation of extracellular matrix (ECM) components in regulating corneal stem cell compartment. Results obtained by various laboratories suggest that the extracellular matrix plays a central role in regulating stem cell commitment, corneal differentiation, and participation in corneal wound healing, in addition to other environmental signals such as cytokines and growth factors. The niche could define cell division patterns in corneal stem cell populations, establishing whether stem cells divide asymmetrically or symmetrically. Characterization and understanding of the factors that regulate corneal epithelial stem cells should open up new paths for developing new therapies and strategies for accelerating and improving corneal wound healing.

## Introduction

Stem cells and their possible therapeutic applications currently constitute an extremely active area of research with the potential to revolutionize medical practice. Despite the apparently recent foundation of the field, its origin dates back to the second half of the 19^th^ century, when the term “stem cell” appeared in the scientific research conducted by the German scientist Ernest Haeckel (1868). Thereafter, German zoologists Theodor Boveri and Valentin Häcker (1892) independently adapted this term to describe the developmental process of the sea urchin and nematode Ascaris [1,2], and the copepod [1,2], respectively. Later, at the end of the 19^th^ century and the beginning of the 20^th^ century, Arthur Pappenheim (1896) and Ernst Neumann (1912) extended the use of the term to designate all precursor cells in the hematopoietic system [1,2]. Nevertheless, for many years stem cells remained ambiguous and theoretical entities, characterized by their self-renewal and differentiation abilities. Consequently, in this early period, the discussion about a tissue’s origin during the emergence or development of cancer mostly focused on embryonic cells or embryonic-like cells, and on changes in cell growth.

The earliest experimental evidence supporting the existence of stem cells was obtained in the 1960s after the self-renewing abilities of bone marrow cells implanted in irradiated mice were analyzed [3,4]. These studies established the first quantitative assay for stem cells, based on the ability of transplanted cells to form colonies, a measure that may reflect their proliferative potential. Subsequently, scientists developed assays based on criteria that must be fulfilled by stem cells. Among these assays, measuring proliferative potential either by determining colony-forming ability [5-7] or serial transfer in cell culture [8,9], as well as retaining DNA precursor analogs due to their slow cell cycling [10-14] became the most powerful tools for localizing and characterizing stem cells. In addition, the expression of specific surface antigens, the lack of terminal differentiation markers [15,16], and higher adherence to certain substrata [17,18], led to methods for enriching and cultivating tissue stem cells.

After evaluating tissues that undergo continuous renewal, authors concluded that adult stem cells have the following specific characteristics: i) self-renewal ability through mitotic cell division, ii) unlimited proliferative potential, and iii) capacity to differentiate into a wide range of specialized cell types [19,20]. Self-renewal is crucial, since it enables stem cells to participate in creating new tissues and, at the same time, guarantees the maintenance of the stem cell compartment. Asymmetric cell division is a possible mechanism involved in self-renewal. Asymmetric cell division maintains adequate numbers of tissue stem cells and results in two unequal daughter cells: one that enters the differentiation process and another that retains stemness properties [21-24].

The following sections discuss the evidence regarding the existence of stem cells in ocular structures, mainly in the corneal epithelium. In addition, they describe the most important characteristics of stem cells’ residence site (the niche), as well as its role in corneal epithelial renewal and wound healing. The purpose of this article is to provide a comprehensive overview of the field. Since this review is not exhaustive, the author expresses his apologies to all the leaders in the stem cell field who were not included in this paper.

### Stem cells in ocular tissues

The study of stem cells has been performed mainly with two kinds of stem cells: embryonic and adult/somatic. Embryonic stem cells originate from preimplantation embryos. In cell cultures, embryonic stem cells can undergo cell division for long periods without differentiating, until they develop into cells and tissues that belong to one of the three primary germ layers.

Adult stem cells locate in specific, protected sites in many organs and differentiated tissues. Most adult stem cells are “tissue-specific,” since they can self-renew and differentiate only into the cell types found in the organ used as the source for the cells.

More recently, after examining the ability of 24 transcription factors to induce and maintain a pluripotent state in mouse and human embryonic or adult fibroblasts, Yamanaka and colleagues found that four factors, Oct-4, Sox2, c-Myc, and Klf4, led to reprogramming of cells transfected with these factors. The cells reached a stem cell-like state [25,26]. This new type of stem cell, called an induced pluripotent stem cell (iPSC), constitutes a promising system with potential applications in regenerative medicine, cell-based therapy, disease modeling, and drug discovery.

Some authors found evidence using experimental approaches such as retention of DNA precursor analogs [11-14] that helped to identify the alleged location of adult stem cells in various tissues: the epidermis [13,14,27,28], prostate [29] and bladder epithelia [30,31], terminal bronchioles [32], liver [33,34], dental pulp [35], intervertebral cartilage [36], bone growth plate [37], and skeletal muscle [38], among others. Although experimental criteria are the basis for the alleged locations of ocular stem cells, in most eye tissues adult stem cells have not been characterized in-depth.

#### Retina

Early studies considered that the mammalian retina lacked stem cells because it was unable to regenerate [39]. Later, von Leitnher et al. proposed that retinal precursors resided in the periphery of the retinal pigmentary epithelium [40]. However, it later emerged that cells from the pigmented ciliary margin develop into spherical colonies, which, after being dissociated into a cell suspension, produced various differentiated retinal cell types [41]. These results supported the possibility that retinal stem cells could reside in the pigmented ciliary margin epithelium.

#### Lens

Based on the distribution of label-retaining cells, authors have proposed that lens stem cells are located at the anterior central region of lens [42]. Nonetheless, Yamamoto et al. [43] analyzed the expression and distribution of proliferation markers such as A1, B1, C, and D1 cyclins, proliferating Cell Nuclear Antigen (PCNA), and 5-bromo-2'-deoxyuridine (BrdU) labeling, and concluded that the germinative zone of the lens epithelium contains transient amplifying cells. In contrast, another author proposed that lens stem cells probably were located in the region immediately anterior to the germinative zone, due to its labeling patterns, and because their response in cell culture was higher than the one observed in cells located on the germinative zone [43]. Nevertheless, since a) the lens is a non-vascularized structure, b) the lens epithelium does not have any protective morphology as observed for other stem cells, and c) the lens does not give rise to any type of tumor cell, Remington and Meyer proposed that lens stem cells reside in the ciliary body [44]. Although evidence from label retention experiments is stronger compared to that obtained from other analyses, the variability observed in the results obtained from different groups led to the conclusion that the location of lens stem cells is still controversial and remains to be elucidated.

#### Corneal endothelium, trabecular meshwork, and stroma

Other presumed locations for stem cells were described for corneal endothelial cells, the trabecular meshwork, and corneal stroma. In the corneal endothelium and the trabecular meshwork, stem cells appear to be located at the transition area (Schwalbe’s ring) between the periphery of the endothelium and the anterior non-filtering portion of the trabecular meshwork [45]. Finally, for the corneal stroma, the stem cell population seems to correspond to the limbal niche cells, which are located at the limbal stroma and participate in vascularization [46].

#### Conjunctiva

For many years, corneal wound healing after injury was explained as a consequence of conjunctival epithelial cell migration and transdifferentiation into corneal epithelial cells [47-50]. However, the incomplete and reversible conversion of conjunctival cells into the corneal epithelium, the recurrent erosions observed in conjunctivalized corneas [51,52], and the discovery of the limbus as the supposed location of corneal epithelial stem cells (see below), led to the conjunctiva being rejected as the source of cells for corneal epithelial healing. Subsequent studies showed that the conjunctival epithelium possesses its own stem cell niche. Label-retention analyses, expression of keratin pairs, and growth potential assays in cell culture suggested that conjunctival stem cells were located at the fornix in rabbits, mice, and humans [53-56]. These results were supported by experiments showing that fornical cells are bipotent, able to differentiate in epithelial and goblet cells [57], and had the most vigorous response to acute and chronic stimulation with tetradecanoyl phorbol myristate compared with palpebral or bulbar conjunctival cells [58]. Currently, the accumulated evidence shows that conjunctival and corneal epithelia constitute two different but contiguous developmental lineages with corresponding stem cell reservoirs.

### Corneal epithelial stem cells

Subsequently, the concept that supported conjunctival epithelium as the possible source by transdifferentiation of corneal epithelial cells prevailed. Immunostaining with monoclonal antibodies made by Tung-Tien Sun’s group against the corneal-specific keratin K3 suggested that corneal epithelial stem cells were specifically located at the basal cell layer of the limbal epithelium: the transition zone between the opaque sclera and the clear cornea [59]. This breakthrough rapidly led to a series of experiments that provided further evidence that supported the limbal epithelium as the location of corneal stem cells: mainly the lack of the K3/K12 keratin pair in limbal basal cells [59-61], the existence of label-retaining cells at this location [62], their higher proliferative potential compared with central corneal cells [63], and their ability to grow in colony-forming assays [64].

Various studies also detected specific molecules as possible markers of the basal limbal epithelial cells. p63, a transcription factor previously proposed as a molecular marker of epidermal stem cells [65,66], showed confined distribution to the limbal epithelium [67]. Similar results were observed for the typical mesenchymal intermediate filament vimentin [68-70], for metabolic enzymes such as α-enolase [71,72], and for α_9_β_1_ integrin [73], a receptor for extracellular matrix (ECM) components such as tenascin-C and EMILIN1, which are involved in corneal epithelial cell adhesion and migration [74].

Many authors provided evidence suggesting that stem cells from adult tissues were in a quiescent state or that stem cells progressed in a slow fashion through the cell cycle. Such characteristics made adult stem cells extremely difficult to detect, unless tissues were exposed to long periods of labeling with DNA precursors. After that process, cells that retained the label (LRC) became evident following a label-dilution period; these cells were considered stem cells [13,14,62]. Slow cycling could be a possible explanation for the enrichment of cell populations with stem cells after treatment with toxic concentrations of 5-fluorouracil [75,76], since rapid proliferating cells would be killed by 5-fluorouracil, while cells with slow proliferation would be less susceptible due to the low incorporation rate of the nucleotide analog.

Nevertheless, using the vital DNA binding dye Hoechst 33342, Richard Mulligan’s group [77] discovered a subset of hematopoietic cells that excluded the DNA stain due to the expression of a multiresistence drug protein that pumps out drugs from cells. This cell population, designed as a side population (SP), was enriched with cells that express hematopoietic stem cell markers; therefore, it was proposed that the side population corresponded to the stem cell population. Later studies assigned this role to the adenosine triphosphate (ATP) binding cassette transporter protein ABCG2 during the efflux of drugs and xenobiotics [78,79] and demonstrated the presence of ABCG2 in most adult [80,81] and embryonic stem cell populations [82,83].

After the limbus was investigated, limbal epithelial stem cells were also observed expressing high levels of ABCG2 [84-87]. Messenger ribonucleic acid and protein also showed the highest levels in the limbus [88]. In view of this, ABCG2 could play a role in protecting corneal stem cells from phototoxicity and various oxidative stress-inducing conditions [89].

Despite the wide variety of molecular markers described for limbal epithelial cells, their use for the specific selection of stem cells has not been as successful as expected. This is explained by the persistence of stem cell markers in the early differentiating cells [90,91]. These cells exhibit intermediate features between stem and committed cells, until the expression of the differentiated phenotype leads to downregulation of stem cell markers [90,91]. Therefore, separating cells with techniques that take advantage of stem cell markers assures only the enrichment of stem cells [92], because the isolated population also includes committed cells that progress through the transient amplification period and generate a set of non-proliferative, terminally differentiated cells [93]. In view of such complexity and of the intrinsic difficulties characterizing the corneal stem cell population, authors have investigated this population through analyzing niches and the regulatory functions exerted by the environment.

### Corneal epithelial stem cells and their niche: Regulation of cell differentiation

As soon as the limbus was assumed to be the location of corneal epithelial stem cells [59], various laboratories analyzed the limbal microenvironment, in addition to searching for molecular markers that could be useful for isolating and characterizing stem cells. Based on these studies, authors proposed that adult corneal stem cells were located at a specific region within the limbus. It is believed that this region, the niche, possesses anatomic and functional dimensions that participate in maintaining “stemness.” This region is characterized by stromal invaginations known in humans as the palisades of Vogt. These papillae-like projections show a distinctive vasculature with radially oriented arterial and venous components [94]. Thus, the palisades of Vogt were suggested as the reservoir that i) protects stem cells from traumatic and environmental insults, ii) allows epithelial-mesenchymal interactions, and iii) provides access to chemical signals that diffuse from the rich underlying vascular network [95-97].

Additional studies demonstrated that the limbus contains a specific anatomic structure that probably provides the microenvironmental characteristics that correspond to the stem cell niche. This structure was designated the limbal epithelial crypt (LEC) [98] or limbal crypt (LC) [99], and consists of a cord or finger of cells that invaginates the limbal stroma from the rete ridges located between the palisades and extends radially the conjunctival stroma [98,99]. The expression of cytokeratin K14 in a similar way as observed for basal cells in the rest of the limbus and the maximal staining for ABCG2 [98] and p63 [99] were some of the criteria that led to the suggestion that corneal stem cells reside at the LEC/LC. Thus far, besides humans and pigs, LEC/LC have not been found in other species [100].

Since tissues with unique cellular properties may synthesize different substrates to which the cells adhere, authors performed the biochemical and immunological characterization of the ECM components associated with corneal tissue. Before the limbus was described as the possible location of corneal stem cells, it was known that corneal ECM constituents changed during development until adulthood in chick, mouse, bovine, and human corneas. Authors described that corneas contained collagen types I–VI [101-104], glycosaminoglycans such as heparin, chondroitin, dermatan, and keratan sulfates [105-109], fibronectin and laminin [110], and hyaluronic acid [111]. These initial evaluations also showed that limbal epithelial cells adhere to a rougher surface, with a more complex arrangement of anchoring fibrils than the one observed in the central cornea [112]. This suggested that limbal cells show a different adhesion capacity compared with the rest of the epithelium, a fact supported by the larger hemidesmosomal area detected in central corneal cells [112], which could also lead to differences in cell motility between the corneal regions being proposed.

To further understand the functional differences between the cornea and the limbus, and therefore, the interaction between epithelial cells and the niche, several authors carefully analyzed the corneal basement membrane components. These studies led first to the recognition that the composition of the basal membrane (BM) between the conjunctival, limbal, and corneal epithelia is heterogeneous [113]. Additional characterization of corneal BM led to controversial results, since some authors reported that the central cornea BM lacks collagen IV [114]; while others reported that collagen IV was found in the limbus and the central cornea [113]. This disagreement was later explained as a consequence of the shift in collagen IV chain isoforms between the limbus and the conjunctiva [115,116]; collagen IV α1(IV) and α2(IV) chains show more intense staining at the corneal limbal border, whereas the α3(IV) chain undergoes an abrupt decrease at the limbus [116,117]. In contrast, collagen types IV (α3-α4 chains) and XII were present in the central cornea [117], although collagen IV (α4 chain) was weakly expressed in this region [116,118].

The differential composition of the limbal BM was extended to other components. α2–α5, β1–β3, γ1–γ3 laminin chains, as well as nidogen-1 and -2, and agrin, were preferentially expressed in the limbal BM [117]. In particular, the limbal BM shows patches of components such as agrin, SPARC/BM-40, tenascin-C, laminin γ3 chain, and versican, which colocalize with ABCG2/p63/K19-positive and K3/Cx43/desmoglein/integrin-a2-negative cell clusters, assumed to be formed by stem and early progenitor cells [116,117]. However, researchers described that BM components such as type XVI collagen, fibulin-2, tenascin-C/R, vitronectin, bamacan, chondroitin sulfate, and versican colocalized with vimentin-positive cell clusters containing putative late progenitor cells [115-117] at the corneal–limbal transition zone. In contrast, type V collagen, fibrillin-1 and -2, thrombospondin-1, and endostatin were almost restricted to the corneal BM [116]; others, such as type IV collagen α5 and α6 chains, collagen types VII, XV, XVII, and XVIII, laminin-111, laminin-332, laminin chains α3, β3, and γ2, fibronectin, matrilin-2 and -4, and perlecan, were uniformly expressed throughout all ocular surface epithelia [116,117].

Together, these results suggested that the BM at the LEC/LC has a specific ECM composition, different from that found in the peripheral and central cornea, probably creating a specialized environment that regulates stem cells and their progeny. This environment should support stemness, by inhibiting the expression of the differentiation process and preserving the proliferative abilities in limbal cells.

Currently, there is a debate about the role of stem cells regarding their interaction with the niche. Are they passive entities that respond to systemic or tissue signals by merely adapting their activity to tissue demands? Alternatively, do stem cells affect the surrounding tissue, with more direct activity on the niche where they reside?

Considering the differential composition between the limbal and central corneal basement membranes, the microenvironment clearly has a tremendous, dramatic effect on corneal epithelial stem cells. Evidence that supports the role of the niche, providing the best examples of the influence of environmental signals on epithelial differentiation, was obtained from recombination experiments. In these studies, murine vibrissae hair follicle stem cells were induced to differentiate into corneal epithelial cells by cultivation in a limbus-specific-like microenvironment [119]. Under such environmental conditions that comprise laminin-5 as a major component, and conditioned medium from limbal stromal fibroblasts, researchers observed that cells isolated from hair follicles formed stratified epithelia that expressed cornea-specific markers such as K12 keratin and the transcription factor Pax6, at the messenger ribonucleic acid and protein levels, while the epidermal specific K10 keratin showed strong downregulation [119]. In other experiments, central corneal epithelial cells from the adult rabbit were recombined with mouse embryonic dermis, leading to the loss of the corneal-specific phenotype accompanied by downregulation of Pax6. The loss of expression of the corneal-specific K3/K12 keratin pair was accompanied by the induction of basal keratinocyte markers such as the K5/K14 keratins and the differentiation into epidermal keratinocytes, including cells with a phenotype that belongs to the hair follicle lineage [120].

Altogether, these experiments emphasize the effects of the microenvironment on the programming of epithelial cells into specific lineages. Since specific signals arising from the basement membrane as well as growth factors and cytokines may regulate cell fate, in the cornea, the decision to leave the stem cell compartment could depend on the ECM composition and structure at the limbus.

**Figure 1:**
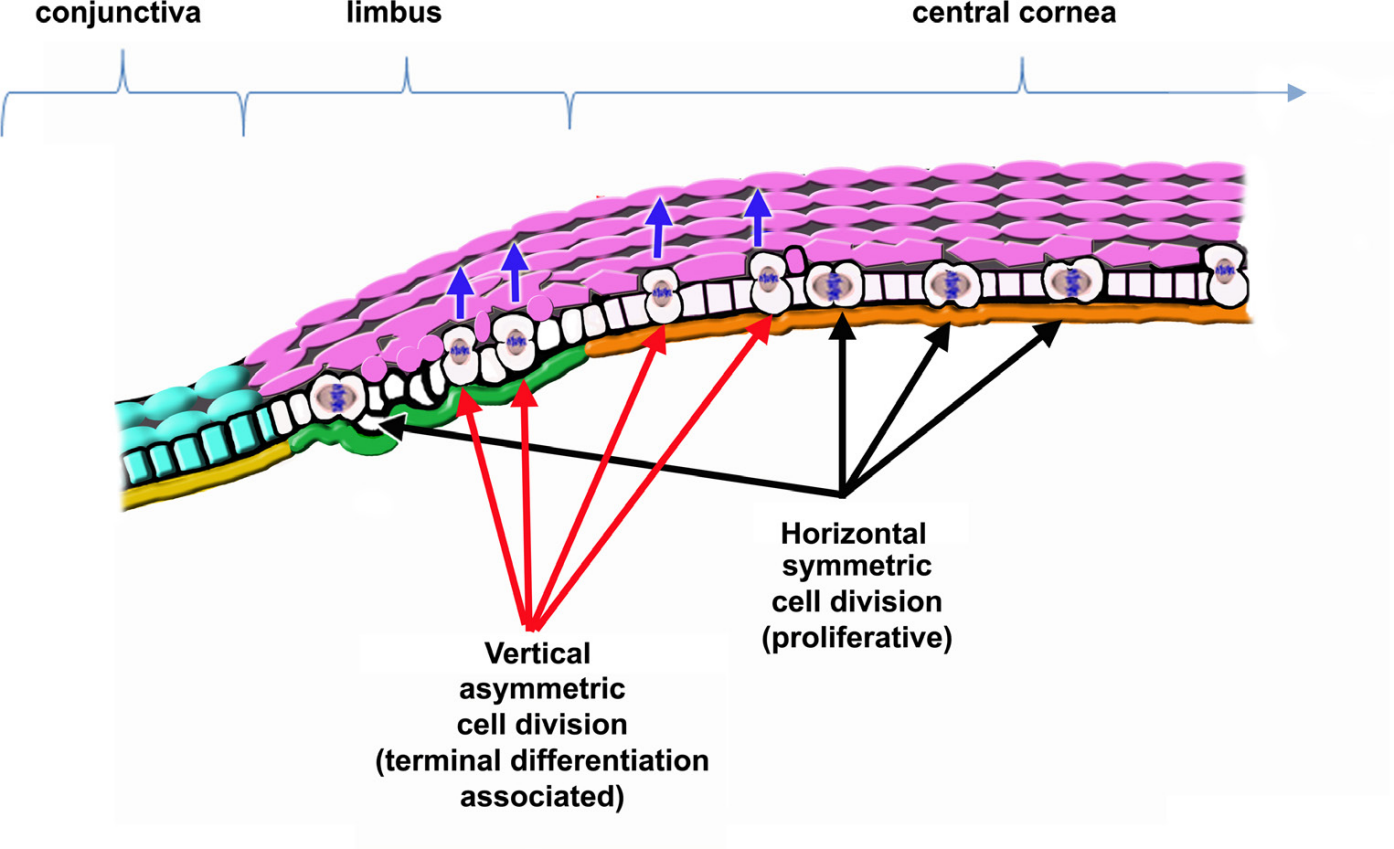
Representation of corneal epithelial cell renewal dependent on proliferative symmetric mitosis of stem cells and their progeny. Stem cells and their progeny proliferate by horizontal, symmetric mitosis; in contrast, asymmetric cell division occurs only in cells that start stratifying and expressing the terminal phenotype. In this case, basal cells that initiate the expression of the terminal phenotype divide with a vertically oriented mitotic spindle. One of the daughter cells remains at the epithelial basal cell layer maintaining its proliferative abilities, and the other leaves the basal layer and enters the suprabasal compartment, becoming bigger, losing proliferative abilities, and becoming terminally differentiated (pink cells). In this model, the basement membrane (BM) modulates the self-renewal and proliferative abilities of stem cells and their progeny based on the its composition and structure. Green=limbal BM. Orange=peripheral and central cornea BM. Yellow=conjunctival BM. Blue arrows=stratification of terminally differentiating cells.

Under such circumstances, corneal epithelial stem cells could follow one of two alternative courses. The first establishes that stem cells and their progeny proliferate through horizontal, symmetric division. This proliferative pattern would be prevalent at the basal layer of the cornea, including the limbus; in contrast, stratification and expression of the terminal phenotype would depend upon vertical asymmetric cell division. Such asymmetric division would result in daughter cells, dissimilar in morphology and proliferative potential; as a result of the division, cells that enter the suprabasal compartment would be bigger and suffer severe restriction in their proliferative abilities, to begin terminal differentiation [121,122] (see Figure 1). In this model, the ECM would modulate the proliferative abilities of basal cells according to their position along the corneal surface (the limbus versus the central cornea) and would control the orientation of the mitotic spindle, being decisive for terminal differentiation. Cells that detach from the basement membrane would be irreversibly committed to express a differentiated phenotype. This possibility is supported by the observation that most basal cells in the corneal epithelium express proteins involved in spindle orientation, such as Partner of Inscuteable (Pins) [123].

**Figure 2:**
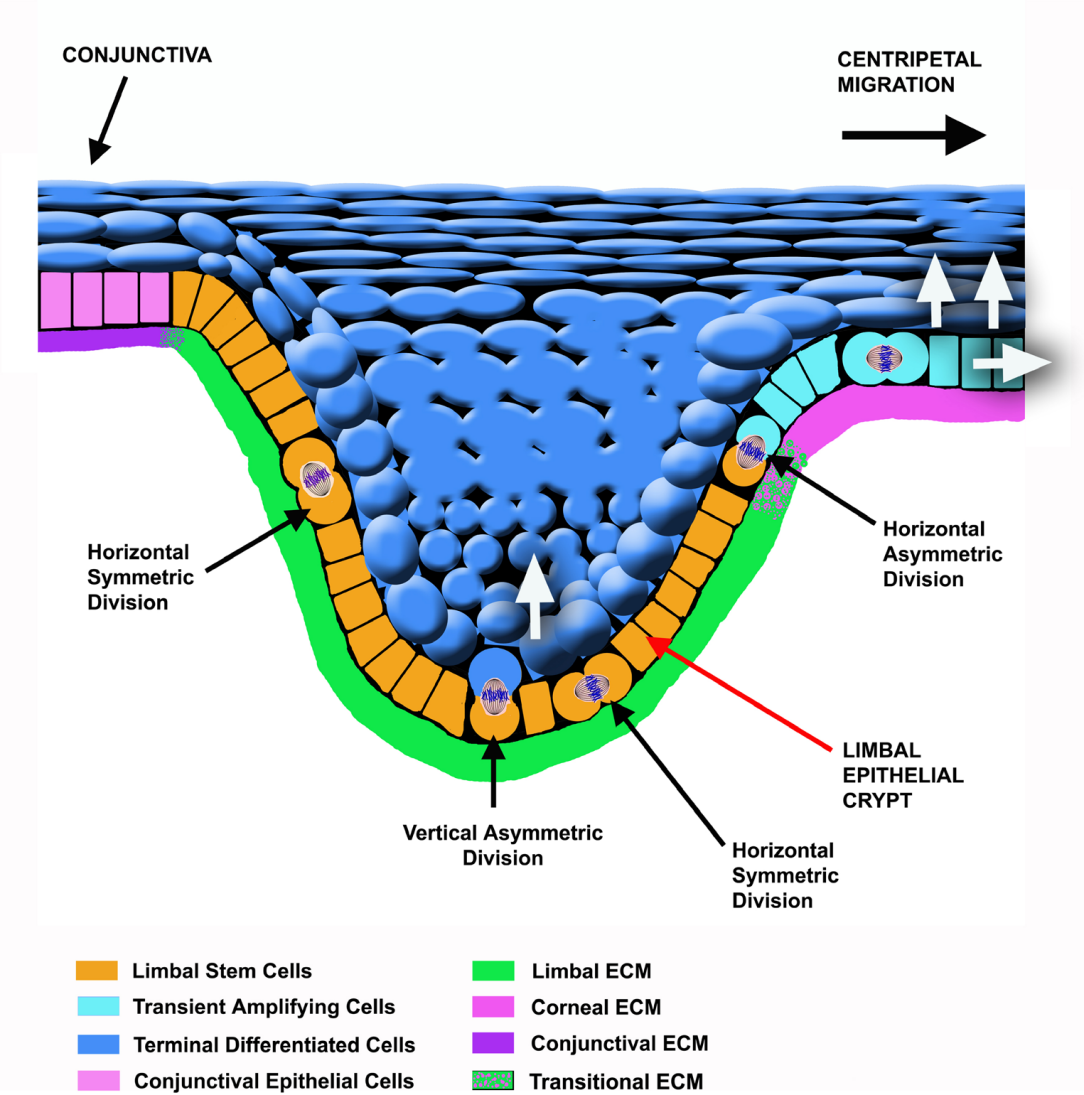
Schematic representation of the limbal epithelial crypt. The extracellular matrix composition and structure may regulate limbal stem cell fate providing information about their position. Depending on the position of cells at the limbal epithelial crypt, the orientation of the mitotic axis during asymmetric cell division of limbal stem cells could be either vertical or horizontal. An asymmetric dividing stem cell would give rise to another stem cell and a transient amplifying basal cell that would migrate to the peripheral cornea when division occurs in the horizontal axis. Conversely, the stem cell could originate another stem cell and a limbal suprabasal differentiated cell when division takes place following the vertical axis; in this case, loss of contact between one of the daughter cells and the basement membrane would determine the initiation of the differentiation process. White arrows indicate the movement of cells after commitment. Differentiation leads to the expression of the terminal phenotype.

In the other pathway, asymmetric cell division is restricted to the limbal stem cells, as proposed for most stem cells [23]. If this is true, the decision to leave the stem cell compartment would depend upon asymmetric division, which would be oriented either horizontally or vertically (Figure 2). Consequently, symmetric cell division would be merely proliferative, and would not be essential for cell commitment. Consequently, the orientation of the mitotic spindle during asymmetric cell division would be defined by extrinsic mechanisms, i.e., the niche or microenvironment in which stem cells reside [124] (Figure 2). To support this proposal, numerous BM components [112,113,115,118,125], as well as growth factors and cytokines such as keratinocyte growth factor [126], interleukin-6 [127], epidermal growth factor, and, fibroblast growth factor β [128], or molecules belonging to the Wnt family [129], among others, show a differential composition or distribution at the limbal, peripheral, and central cornea. Together, they may be involved in establishing the corneal niche.

According to the second model, stem cells in the limbus undergo either vertical or horizontal asymmetric mitosis during corneal replenishment or during wound healing. After asymmetric cell division, one of the daughter cells loses contact with the limbal BM either by moving into the suprabasal cell layers or by moving and proliferating into the central cornea, and initiates the differentiation process [124] (Figure 2). When this event occurs, daughter cells also become regulated by the components of the central cornea basement membrane and growth factors such as insulin-like growth factor 1 [128], or molecules of the Wnt family such as Wnt3, Wnt7a, Wnt7b, and Wnt10a, which are upregulated in the central cornea [129].

It is still unknown how limbal stem cells influence the surrounding cells, tissues, and organs, and, therefore, the way in which they can modify their niche. Although there is some evidence regarding the participation of the family of Notch receptors and their associated signal transduction pathway [130-133], more knowledge is needed about how limbal cells interact with the niche to regulate and enhance responses involved in maintaining and repairing tissue.

### Corneal epithelial stem cells: Renewal and wound healing

Beginning with the discovery of the centripetal cell migration that occurs in the cornea, early studies on epithelial cell renewal led to the conclusion that the proliferative source of the corneal epithelium resided at its basal cell layer and at the corneal periphery. In these experiments, authors showed that two separate processes participate in renewing the corneal epithelial cells: i) the division of basal cells, mainly at the corneal periphery, with their successive movement into suprabasal cell layers, and ii) the progression of cells across the limbus toward the center of the cornea, before superficial cells are desquamated [134-136].

Later, as previously discussed, it was proposed that the presumptive location of corneal epithelial stem cells was the limbus. Accordingly, the corneal epithelium consists of stratified tissue with a high self-renewal rate based on the regenerative capacities of the stem cells located at the basal layer of the limbus and the proliferation of basal cells from the central cornea [59,62]. In such well-structured tissue, suprabasal cells at the limbus and at the central cornea undergo terminal differentiation and lose their proliferative abilities. While basal cells located at the central cornea proliferate actively, basal cells at the limbus consist of a mixture of slow-cycling stem cells and cycling transient amplifying cells [62,93].

As stated by this hypothesis, the normal corneal epithelium remains in a steady-state in which cell proliferation is necessary only for replacing cells lost by terminal differentiation and desquamation. Stem cells located at the LECs divide occasionally [59,63,93,124], and subsequently, their progeny leaves the niche, while undergoing the transient amplification process, which occurs at the basal cell compartment of the peripheral and central cornea [59,63,93,124]. Such transient amplification would imply a gradient or hierarchy of cells with a decreasing proliferative potential along the central cornea [63,93], and comprises a still unknown number of cell divisions, mainly modulated by growth factors and cytokines [137-139] before cells become post-mitotic and begin to stratify.

After wound damage, trauma, or exposure to tumor promoters such as tetradecanoyl phorbol myristate, the tissue response consists of a rapid 8–9-fold rise in the proliferative activity at the limbus, which then is reduced to pretrauma levels after 36–48 h as well as a prolonged twofold increase in proliferation at the peripheral/central cornea that returns to basal levels after the wound closes [58,62]. These results have been interpreted as a consequence of the recruitment and multiplication of the limbal stem cells, and the transient multiplication of the peripheral and central cornea basal cells, respectively [93,140].

This possibility is supported by several lines of evidence that suggest corneal stem cells reside at the limbus: i) mainly the lack of adequate healing of wounds in corneas in which the limbus has been damaged or surgically removed [141-143], ii) limbal transplantation to restore wound repair [144], or iii) the presence of holoclone-forming cells in limbus but not in the central cornea [53,63], among others. So, the reader may ask, what is the role of the niche in corneal wound healing? The answer is mostly unexplored. However, results from various groups suggest that the niche rules stem cell behavior by regulating the cell division pattern, in part through the active role of basement membrane components at the limbus. Recent results that strongly support that in the adult corneal epithelium asymmetric divisions may occur only at the limbus [124], together with evidence that restricts the expression of specific markers and the expression of cell proliferation and cell fate regulators such as ΔNp63α [145] and Notch1 [146] to stem cells, suggest that asymmetric cell division is part of the differentiation program in corneal epithelial cells [147]. Therefore, the basement membrane would provide limbal stem cells with information about their position and fate. Thus, depending on the position of cells at the limbal epithelial crypt, the orientation of the mitotic axis during asymmetric cell division of limbal stem cells could be either vertical or horizontal. Consequently, an asymmetric dividing stem cell would give rise to another stem cell and either a transient amplifying basal cell located at the peripheral cornea (when the division occurs in the horizontal axis) or a limbal suprabasal differentiated cell (when the division takes place following the vertical axis).

Accordingly, corneal wound healing should elicit a tissue response in which limbal stem cells undergo a few cell cycles and give rise to numerous transient amplifying cells that constitute the migratory/proliferative edge of the wound. The size of the transient amplification of early precursors and committed cells would then be modulated by changes in the ECM composition and ECM receptors during corneal wound healing [148-150], and by changes in the expression of growth factors such as insulin-like growth factor 1 [128], epiregulin [151], or stem cell factor (c-kit ligand) [152].

Since growth factors and ECM components regulate the migration and proliferation of the transient amplifying cells, with the preceding proliferation of limbal stem cells, growth factors and the ECM could be used alone or combined, to accelerate and improve repair of corneal wounds, and reduce consequences associated with corneal damage. Examples of this approach include the application of growth factors to promote corneal wound healing such as epidermal growth factor [153,154], basic fibroblast growth factor [155], tumor necrosis factor α and interleukin-1 [156], and ECM components such as decorin [157].

Although some results have suggested that treating corneal wounds with growth factors or ECM components offers new opportunities for therapeutic intervention, some evidence implies the need for a complex set of growth factors and ECM components, perhaps in a specific three-dimensional arrangement, to improve and accelerate corneal wound healing. This possibility is supported by the application of cultured epidermal sheets as temporary wound coverings on experimental excimer laser corneal ablations. These epidermal sheets increase the reepithelialization rate of wounds by about 60%, in addition to reducing inflammation and scarring at the wound site [158]. Such corneal healing improvement has been explained through the synthesis and release of growth factors, cytokines, and ECM onto the wound bed by cultured epidermal sheets [159]. A similar mechanism for enhancing wound healing could occur during treatment of corneal wounds with amniotic membranes [160].

### Conclusion

Thus far, the study of limbal stem cells and their regulation by environmental signals, either cytokines, growth factors, and their interaction with other cell populations, is almost unexplored. Researchers have identified a set of molecular markers that may be used for enriching stem cells in isolated populations; however, this analysis led to the conclusion that there is no specific, unique marker for identifying and isolating limbal stem cells. In spite of these difficulties, this collection of markers allowed the characterization of the stem cell niche, and demonstrated that the limbus shows special characteristics, in composition and/or structure, that make it different from the peripheral and central cornea.

This evidence, together with cell culture and clonal assays, suggests that the corneal epithelial cells comprise two different populations: stem cells and transient amplifying cells. The latter corresponds to the progeny of the stem cells, which possesses limited proliferative potential and it is probably committed to terminal differentiation. The number of cell cycles undergone by transient amplifying cells depends on stimuli from the environment.

Although numerous studies indicate that corneal epithelial stem cells reside preferentially at the basal layer of the limbal zone rather than uniformly in the entire corneal epithelium, recent results suggest that corneal stem cells may also be at the central cornea [161]. In spite of the controversial nature of these results, they bring up many questions about the possible function of corneal stem cells during tissue renewal or their migratory potential from the limbus. In either case, a major question involves the possible conditioning effect of stem cells upon the environment: Can stem cells modify their surroundings to form new niches? The possible location of epithelial stem cells in the central cornea could help explain the transdifferentiation of the adult corneal epithelium when it receives signals from embryonic dermis [120], unless researchers could demonstrate that expression of the corneal epithelial phenotype is reversible by stimulation of the appropriate signaling pathways. Understanding of the niche’s biologic activity on stem cells may lead us to develop new therapies for accelerating and improving corneal wound healing.
